# Role of the novel HSP90 inhibitor AUY922 in hepatocellular carcinoma: Potential for therapy

**DOI:** 10.3892/mmr.2015.3725

**Published:** 2015-05-04

**Authors:** WEI CHENG, AIMUDULA AINIWAER, LEI XIAO, QIAN CAO, GE WU, YING YANG, RUI MAO, YONGXING BAO

**Affiliations:** Department of Oncology, The First Affiliated Hospital of Xinjiang Medical University, Urumqi 830011, P.R. China

**Keywords:** hepatocellular carcinoma, heat shock protein 90, microvessel density, AUY922, targeted therapy

## Abstract

The aim of the present study was to determine the correlation between hepatocellular carcinoma (HCC) and heat shock protein 90 (HSP90), involved in tumor angiogenesis, and to evaluate the effect of AUY922, a HSP90 inhibitor, in HCC. The expression of HSP90 and microvessel density (MVD) were measured in tissue samples from 76 patients with HCC by immunohistochemistry. Western blot analysis was performed to detect the expression of HSP90 in the HCC tissues and different HCC cell lines. The effects of time and concentration treatment with the AUY922 HSP90 inhibitor were investigated in HepG2 cells. Cell proliferation was measured using an MTT assay and a Transwell assay was performed to evaluate the migration of the HepG2 cells following treatment with different concentrations of AUY922. Positive staining of HSP90 was observed in 88.16% (67/76) of the HCC tissues, compared with 16.67% (4/24) of the normal tissues. The difference in the expression of HSP90 between the HCC and normal tissues was statistically significant (P<0.001). Tumors exhibiting positive expression of HSP90 had significantly higher MVD compared with the HSP90-negative counterparts (82.8±12.44 vs. 23.8±8.07, respectively; P<0.001). The expression levels of HSP90 were positively correlated with MVD in all the tissue samples (r_s=0.724; P<0.001). AUY922 inhibited the proliferation of the HepG2 cells in a time- and concentration-dependent manner, and the migration of HepG2 cells was distinctly suppressed following treatment with AUY922. These data suggested that the angiogenesis of human HCC may be mediated by HSP90, and that the specific HSP90 inhibitor, AUY922, has a therapeutic role in the treatment of HCC. Therefore, HSP90 may represent a selective target in molecularly targeted treatment of HCC.

## Introduction

Hepatocellular carcinoma (HCC) is one of the most malignant types of cancer and is listed as the second most frequent cause of cancer-associated mortality in males, and the sixth most frequent cause in females worldwide (1). Surgical resection is the most effective way of treating HCC, however, the majority of patients present at an advanced, inoperable stage. These patients have a poor prognosis and require other types of treatment (2). Palliative treatments, including chemotherapy and radiation therapy only contribute to 6% of the overall 5-year-survival rate in HCC (3). Therefore there is an urgent requirement to develop novel therapeutic strategies for HCC.

It is understood that solid tumors cannot continue to grow without the formation of new vessels (4). Unlimited tumor expansion requires continuous angiogenesis to acquire sufficient oxygen and nutrients. Angiogenesis, the formation of new blood vessels from the original vasculature, is a complicated multi-step process, which involves a number of signal transduction pathways (5). Tumors secrete a variety of pro-angiogenic factors, among which, vascular endothelial growth factor (VEGF) has been investigated in the most depth for its association with tumor angiogenesis (6). VEGF binds to vascular endothelial growth factor receptor (VEGFR), usually VEGFR-2, and activates receptor tyrosine kinase. This causes a signal transduction series, which induces and promotes endothelial cell proliferation and migration, respectively, eventually leading to neovascularization (6). Microvessel density (MVD) is considered a golden standard in evaluating tumor angiogenesis (7). To quantify the angiogenic status, markers of endothelial cells, including Factor VIII, CD31 and CD34, have been used (8). With the identification of novel biological markers of cancer, molecular targeted therapies are considered the most promising strategies for the management of patients with progressive HCC.

Heat shock protein 90 (HSP90) is a molecular chaperone, which comprises 1–2% total cellular protein content and regulates the correct conformation, activity, function and stability of >200 client proteins (9). A variety of receptor tyrosine kinases, including VEGFR, insulin-like (I)GFR, and epidermal GFR are client proteins of HSP90, which depend on HSP90 to achieve active conformation or to increase stability (9). The classic HSP90 inhibitors are benzoquinone and ansamycins, including geldanamycin and its derivative 17-allylamino-17-demethoxygeldanamcyin (17-AAG). The inhibition of HSP90 has been achieved using the novel, low molecular weight, adenosine triphosphate (ATP)-competitive non-geldamycin HSP90 inhibitor, AUY922. This compound has been considered to offer advantages over ansamycin and benzoquinone HSP90 inhibitors, including 17-AAG, based on independence from the metabolism of NAD(P)H quinone oxidoreductase 1, expression of P-glycoprotein expression and favorable aqueous solubility (10,11). In the present study, the expression of HSP90 and MVD in HCC was investigated, and the antitumor efficacy of the novel AUY922 HSP90 inhibitor, AUY922, in inhibiting HCC cell proliferation and migration was evaluated.

## Materials and methods

### Patients and tissue samples

The present study was approved by the ethics committee of the First Affiliated Hospital of Xinjiang Medical University (Urumqi, China). The pathology specimens and medical records were reviewed from the First Affiliated Hospital of Xinjiang Medical University database. The preoperative diagnosis was based on the clinical history, symptoms, signs, endocrine evaluation, imagine examination, including magnetic resonance imaging and computed tomography. Paraffin-embedded pathological specimens from 76 patients (51 males/25 females; mean age, 59.57±9.16 years old; age range between 42 and 69 years) with HCC were obtained from the archives of the Department of Oncology, First Affiliated Hospital of Xinjiang Medical University between 2010 and 2013. Adjacent non-tumorous liver tissues were obtained from 12 of the patients (5 cm to the tumor area), to serve as normal controls. The patients involved in offering samples for the investigation signed informed consent forms.

### Immunohistochemical analysis

To evaluate the expression levels of HSP90 and MVD in ACC, immunohistochemical analyses were performed using an EnVision method (DAKO, Glostrup, Denmark). Antigen retrieval was achieved by microwave at 750 W for 15 min, and the sections were incubated with 10% normal goat serum at room temperature for 10 min to block non-specific reactions. This was followed by washing with phosphate-buffered-saline (PBS) and incubation with polyclonal mouse anti-human HSP90 antibody (Abcam, Cambridge, MA, USA) diluted to 1:100 for 12 h at 4°C. CD34 was a monoclonal mouse anti-human antibody (Novocastra Laboratories, Ltd., Newcastle-Upon-Tyne, USA), diluted to 1:200. The positive controls were gastric carcinoma and colon carcinoma, with positive expression levels of HSP90 and CD34. PBS was used instead of the primary antibodies as a negative control. The localization of immunostaining was demonstrated by incubation with the EnVision-peroxidase system.

### Evaluation of the immunohistochemical results

The immunohistochemical analyses designated a result as positive for HSP90 if purple-brown granules were located diffusely in the cytoplasm of the tumor cells. The lack of any purple-brown or brown-red pigmentation in the cytoplasm of a tumor cell was considered negative (12). For HSP90 semi-quantitative immunoanalysis, the percentage of positive staining in the tumor cells was determined. A total of five representative high-power fields were selected, and the number of positive-staining cells was calculated. The following categories were used: Negative (−); weak (+)=1–10%; moderate (++)=11–50%; strong (+++) ≥51%. The results were scored by two independent pathologists in a blinded-manner into tumor subtypes. A single microvessel was defined as any brown or brownish yellow CD34-immunostained endothelial cell. The MVD was evaluated using a method previously described by Weider (7): High vascular density areas were selected under a low power objective and the numbers of vascular cells stained with CD34 were determined in three visual fields under a high power microscope (magnification, ×400). The average value was regarded as the MVD value of the tumor sample.

### Cell culture and reagents

A total of five HCC cell lines, SK-Hep1, HepG2, HEK-293T, HCCLM3 and HuH7, and one normal liver cell line (L-02) were obtained from American Type Culture Collection (ATCC, Manassas, VA, USA) and were maintained in a humidified atmosphere containing 5% CO_2_ at 37°C, which were cultured and passaged in RPMI-1640 medium (Hyclone, Thermo Fisher Scientific, Logan, UT, USA) containing 10% fetal bovine serum (FBS; Invitrogen Life Technologies). The cells at logarithmic growth phase were harvested for subsequent experiments once the cells had reaced 80% confluence. The HCC cell lines were maintained in Dulbecco’s modified Eagle medium (DMEM) (Invitrogen, Life Technologies) supplemented with 10% FBS, 100 U/ml penicillin G and 100 mg/ml streptomycin sulphate (Sigma-Aldrich, St. Louis, MO, USA). The AUY922 HSP90 inhibitor was purchased from Selleck (Houston, TX, USA). The compounds were dissolved at 10 mM in dimethylsulf-oxide (DMSO) at stock solutions and stored at −20°C. Mouse anti-human HSP90 antibody was obtained from Abcam.

### Western blot analysis

Western immunoblot analyses were performed with protein lysates obtained from snap-frozen HCC tissue samples and cell lines. The protein levels were determined using a Bicinchoninic Acid Protein Assay kit (Pierce Biotechnology, Inc. Rockford IL, USA). The respective tissue protein (30 *µ*g) were separated by 10% SDS-PAGE (using 10% gels) and transferred onto polyvinylidene fluoride membranes (Millipore, Billerica, MA). The membranes were blocked with 5% nonfat milk and then incubated with monoclonal mouse anti-human HSP90 polyclonal antibody (1:100; cat. no. ab13492; Abcam) and monclonal mouse anti-actin (1:10,000; cat. no. 0869100; MP Biomedicals, Santa Ana, CA, USA). The membranes were washed three times for 10 min each with Tris-buffered saline (50 mM Tris, pH 7.4, 0.9% NaCl) containing 0.05% Tween-20 (TBS-T; Biohao, Beijing, China) and incubated with phycoerythrin-conjugated secondary antibodies (1:2,000; donkey anti-mouse IgG H&L; cat. no. ab7003; Abcam). Membranes were then washed again three times for 10 min each with TBS-T. The target protein bands were visualized using the Pierce enhanced chemiluminescence system (Pierce; Thermo Fisher Scientific, Waltham, MA, USA). All western blot analyses were performed three times.

### Cell viability measurement

The viability of the cells was analyzed by Thiazolyl blue (MTT; Sigma-Aldrich). The MTT assay examines the activity of metabolic enzymes in the mitochondria of live cells. Therefore MTT can reflect cell proliferation. Cells that were grown to 70–80% confluency in 96 well plates were treated with AUY922 at a final concentration of 1, 2.5, 5, 10, 25, 50, 100 nM for 24, 48 or 72 h, respectively. Cells treated with 5 mg/l cisplatin (DDP) were used as positive control. After the reaction with the drugs for 24, 48, 72 h, cells were then treated with MTT (10 ml/well) for 4 h at 37°C. The cells were subjected to an absorbance reading at 570 nm using a 96-well microplate reader (CKX41SF; Olympus, Tokyo, Japan). The optical density (OD) values were normalized to those of the cells treated with 0 nmol/l AUY922. The percentage of residual cell viability was determined as [(OD experiment group − OD blank group) / OD negative group − OD blank group)] ×100%. Each assay was performed three times. In order to calculate IC_50_, a dose-responsive curve was fitted using a nonlinear regression model with a sigmoidal dose response. The IC_50_ was automatically produced by GraphPad Prism 5.0 (GraphPad Software, Inc., La Jolla, CA, USA).

### Cell migration assay

The motility capabilities of the cells *in vitro* were measured using Transwell chambers (Corning, Corning Incorporated, Corning, NY, USA). Subsequently, four groups of cells (5×10^5^) were seeded on the upper wells with serum-free medium. Medium with 20% FBS was plated in the bottom wells as chemoattractants. After 48 h incubation, the cells were fixed with 90% methanol and stained with 1% crystal violet (Santa Cruz Biotechnology, Inc., Dallas, TX, USA) for 30 min at 37°C. Cells staying on the upper side of the membranes were wiped, while those on the lower side were counted and photographed with microscope.

### Statistical analysis

Data analyses were performed using SPSS statistical package 15.0. Phenotypic differences in quantitative traits were assessed by genotype using Student’s t-test or analysis of variance. Differences in the distribution of qualitative traits by genotype were assessed using standard χ^2^-square analysis and Fisher’s exact test. To evaluate the correlation of HSP90 and MVD, Spearman’s correlation test was used. P<0.05 was considered to indicate statistically significant difference.

## Results

### HSP90 is overexpressed in HCC tissues compared with normal tissues

**Figure 1:**
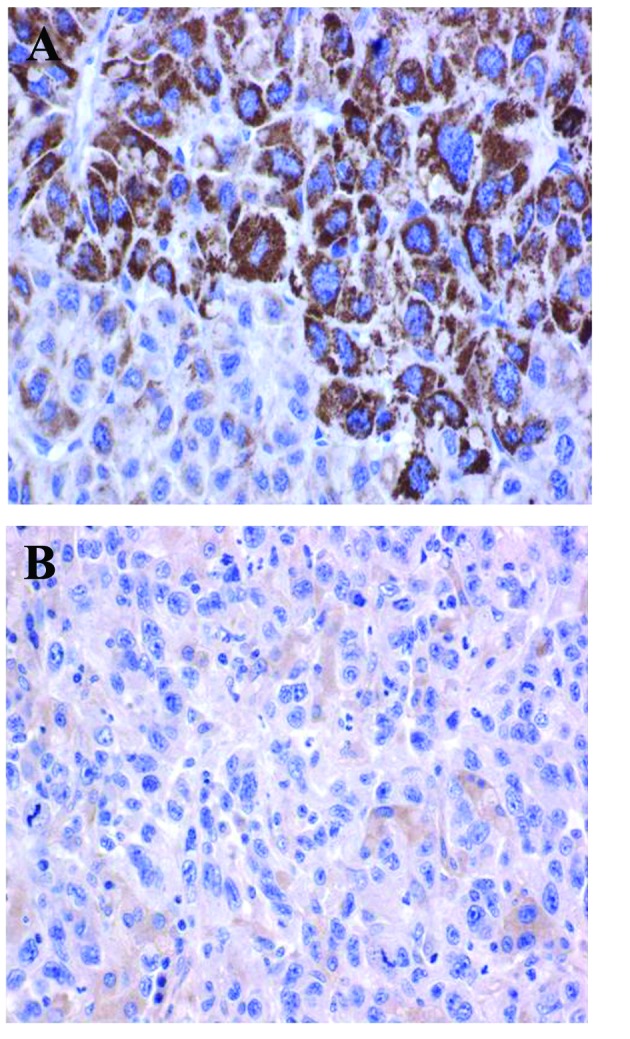
(A) Positive expression of HSP90 in HCC tissue (magnification, ×40); (B) Negative expression of HSP90 in the adjacent non-tumorous liver tissue (magnification, ×40). HCC, hepatocellular carcinoma.

**Figure 2:**
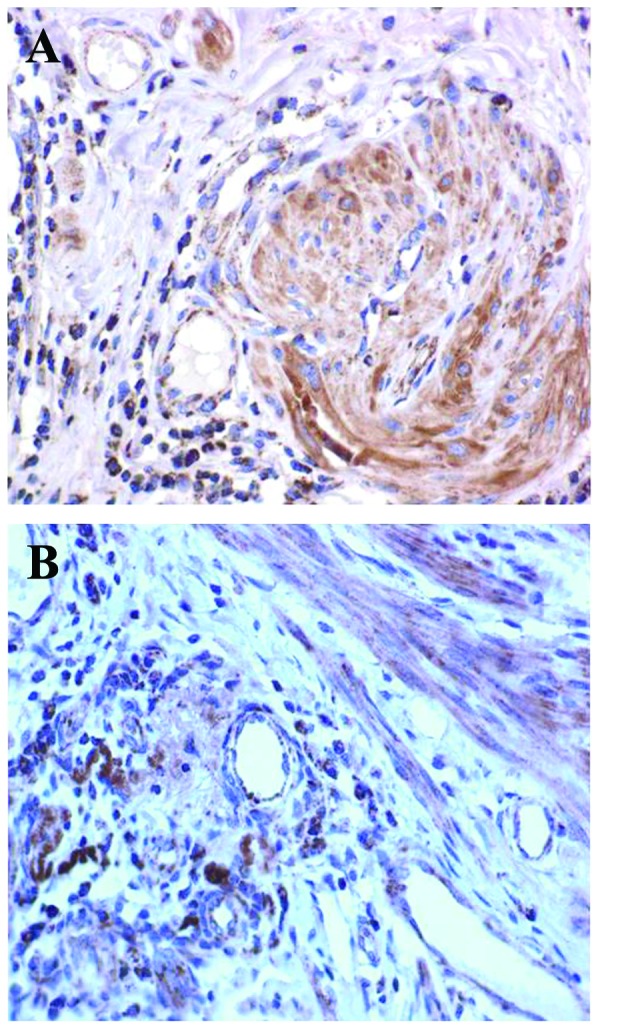
(A) Positive expression of MVD, observed following CD34 immunostaining, in HCC tissue (magnification, ×40). (B) Negative expression of MVD observed following CD34 immunostaining in the adjacent non-tumorous liver tissue (Envision, ×40). MVD, microvascular density; HCC, hepatocellular carcinoma.

**Figure 3:**
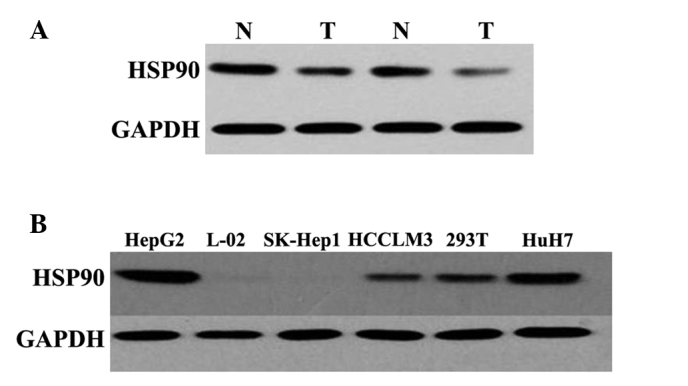
(A) Western blot of the expression of HSP90 in the HCC tissues and in the adjacent normal adrenal tissues; (B) Western blot of the expression of HSP90 in different HCC cell lines. HCC, hepatocellular carcinoma. HSP90, heat shock protein 90; N, normal tissue; T, tumor tissue.

The expression of HSP90 was higher in 67 of the 76 randomly selected positive human HCC tissues compared with the adjacent normal tissues. Staining of HSP90 was observed in 88.16% (67/76) of the HCC tissues, compared with 16.67% (4/24) of the normal tissues, and this difference between the expression of HSP90 between the HCC and normal tissues was statistically significant (P<0.001). Representative images of the HSP90 and MVD are shown in Figs. 1 and 2. The tumor tissues with positive expression of HSP90 had a significantly higher MVD compared with their HSP90-negative counterparts (82.8±12.44 vs. 23.8±8.07, respectively; P<0.001). The expression levels of HSP90 were positively correlated with MVD in all the samples (r_s=0.714; P<0.001). A total of 30 tissue samples of HCC and adjacent normal tissues were examined to detect the protein expression of HSP90 using western blot analysis. The protein expression level of HSP90 was also overexpressed in the HCC tissues compared with the normal tissues, and the difference between the two groups was statistically significant (P<0.01; Fig. 3A). In addition, the expression of HSP90 was detected in five HCC cell lines and one normal liver cell using western blot analysis, the results of which revealed that the expression of HSP90 was high in the HepG2 cells and low in the HuH7 cells(Fig. 3B). Therefore, the HepG2 cell line was selected for use in the subsequent investigations.

### HSP90 inhibitor, AUY922, has an inhibitory role in the proliferation of HepG2 cells

**Figure 4:**
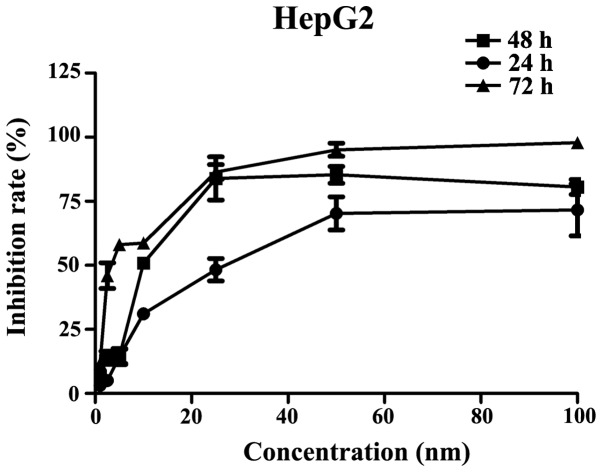
AUY922 inhibits the proliferation of HepG2 cells. The number of cells were measured using an MTT assay. AUY922 inhibited HepG2 cell proliferation at 24, 48 and 72 h, in a concentration-dependent manner. Data represents the mean ± standard error. HCC, hepatocellular carcinoma.

The HepG2 cells were treated with 1, 2.5, 5, 10, 25, 50, or 100 nM AUY922 for 24, 48 or 72 h, and cell proliferation was measured using an MTT assay. As shown in Fig. 4, the MTT assay demonstrated that AUY922 significantly inhibited cell proliferation in a time- and concentration-dependent manner. A AUY922 concentration of 50 nM exhibited similar efficacy as 5 mg/l DDP. The half maximal inhibitory concentrations at 24, 48, and 72 h were 27., 10.1 and 3.4 nM, respectively.

### Inhibition of the migration of HepG2 cells by AUY922

**Figure 5:**
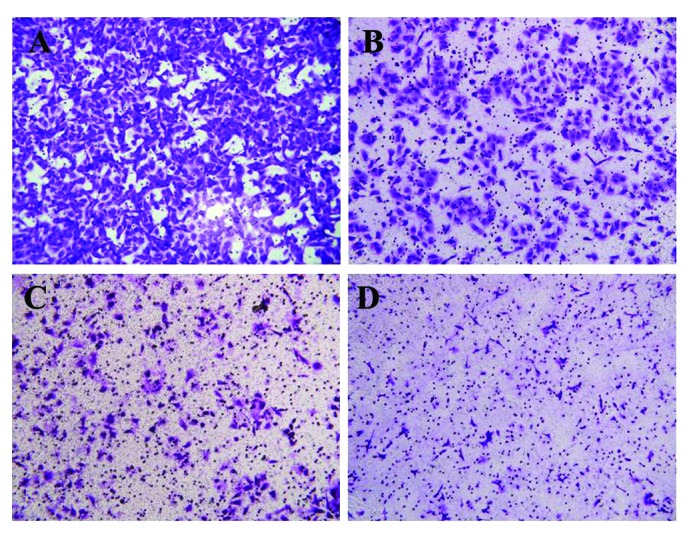
Inhibition of HepG2 cell migration by AUY922. (A) Negative control; (B) 10 nM AUY922; (C) 50 nM AUY922; (D) 100 nM AUY922 (magnification, x20).

A Transwell assay was used to verify the effect of AUY922 on the migration of HCC cells *in vitro*. The results of the migration assay demonstrated that the number of HepG2 cells, which penetrated through the membrane in the AUY922-treated groups was significantly lower than that observed in the negative control group, and fewer HepG2 cells penetrated through the polycarbonate membrane in the 100 nM group compared with the 10 nM group (P<0.05; Fig. 5).

## Discussion

HCC is the sixth most common type of malignancy worldwide and is the third most common cause of cancer-associated mortality (1). HCC is a highly vascularized tumor, which emphasizes the importance of investigating its angiogenesis. Angiogenesis, an indispensable step in the progression of a variety of solid tumors, is a key event in numerous pathological and physiological conditions (5). MVD has been regarded as a gold standard in assessing significant angiogenesis in tumors (7). The MVD in tumor tissues is determined by evaluating tumor-derived vascular endothelial cells using monoclonal antibodies, including CD-31, CD-34 and FactorVIII (13). One of the most potent endothelial mitogens and mediators of angiogenesis is VEGF, and the induction of VEGF in cancer cells can be mediated through activation of various signaling pathways, including phosphoinositide 3-kinase (PI3K)/Akt (14).

HSP90 is the most abundant cytosolic HSP and regulates the maturation and stability of various proteins, which are essential for multiple cell signaling processes (15). In several types of cancer, including breast cancer, non-small-cell lung cancer, and prostate cancer, HSP90 is overexpressed and may contribute to tumour cell survival by mediating the maturation and stability of a variety of client proteins, including the IGF1 receptor and elements of the PI3/Akt, signal transducer and activator of transcription 3 and mitogen-activated protein kinase signalling pathways (15,16). These client proteins of HSP90 are available to promote growth factor independence, resistance to drugs, proliferation, tissue invasion, metastasis and angiogenesis, which are all critical for tumor progression and survival (17). The aim of the present study was to investigate the expression and function of HSP90 in HCC to examine the effects on the oncogenetic process. The expression of HSP90 and the MVD were measured in tissue samples from 76 samples of HCC tissue and 24 samples of adjacent normal hepatic tissues using immunohistochemistry. The results demonstrated positive staining of HSP90 in 88.16% (67/76) of the HCC tissue samples, compared with 16.67% (4/24) of the normal tissue samples, and this difference in the expression of HSP90 between the HCC and normal tissues was statistically significant (P<0.001). The tumors exhibiting positive expression of HSP90 had a significantly higher MVD than their HSP90-negative counterparts (82.8±12.44 vs. 23.8±8.07, respectively; P<0.001). The expression of HSP90 expression was positively correlated with MVD in all the specimens (r_s=0.724; P<0.001). Therefore, a statistically significant correlation was observed between HSP90 and MVD in HCC. Accordingly, the expression levels of HSP90 in five HCC cell lines and one normal liver cell was detected using western blot analysis. It was hypothesized that HSP90, involved in angiogenesis, may be a potential molecular target for the treatment of HCC.

The inhibition of HSP90 has become one of the most popular areas of investigation (18). A previous found that, in cancer cells, HSP90 exhibits higher binding affinity for 17-AAG exclusively, and forms 17-AAG-sensitive HSP90-containing ‘superchaperone’ complexes in malignant cells, whereas normal cells, with predominantly uncomplexed HSP90, are significantly less sensitive to these types of inhibitors (19). By contrast, HSP90 inhibitors preferentially accumulate in tumor cells rather than normal cells (20), and the client proteins, which are most sensitive to HSP90 inhibition are preferentially expressed in tumor cells. Nguyen *et al* (21) observed H358 cell and rat tumor cell lines *in vitro* and demonstrated that 17-AAG markedly inhibits the production of VEGF, which confirmed that 17-AAG is effective in regulating the expression of the genes of VEGF. Hur *et al* (22) also demonstrated that 17-AAG can reduce the angiogenesis of tumor cells by inhibiting the genes of VEGF.

HSP90 inhibitors have improved considerably. AUY922 is part of the isoxazole HSP90-inhibitor family and is a non-geldanamycin analog, which offers prolonged target inhibition and has not been associated with the same degree of hepatotoxicity as its geldanamycin counterparts (23). AUY922 exerts its effects by binding to the ATPase domain of the HSP90 N-terminal, preventing HSP90 from its chaperone functions. This leads to the proteasomal degradation of several relevant client proteins (11). Single agent AUY922 has been observed to exhibit potent preclinical anticancer activity *in vitro* and *in vivo* against a range of histologic cell types, including head and neck squamous cell carcinomas, pancreatic, prostate, lung, cervical, colorectal and breast carcinomas, myelomas and melanomas (24–28). In the present study, HepG2 cells were treated with 1, 2.5, 5, 10, 25, 50, or 100 nM AUY922 for 24, 48 or 72 h. The cell survival rate was measured using an MTT assay to assess the inhibitory effect of AUY922 on the HCC cells. The results revealed that AUY922 significantly inhibited the proliferation of the HepG2 cells in a time- and concentration-dependent manner. AUY922 can inhibit proliferation in various types of tumor cells, according to previous reports (26), therefore, the results of the present study confirm HSP90 as an important target in HCC. The presumptive tumor suppressor function of AUY922 in human HCC was further investigated using a Transwell assay. The results demonstrated that the migratory ability of the cells was significantly suppressed by AUY922, with fewer HepG2 cells penetrating the polycarbonate membrane in the AUY922-treated group compared with the negative control group. This occurred in a dose-dependent manner.

In conclusion, the results of the present study demonstrated that HSP90 was overexpressed in HCC, and that the HSP90 inhibitor, AUY922 inhibited HepG2 cell proliferation and migration in a time- and dose-dependent manner. Treatment involving the inhibition of HSP90 may provide a promising strategy for antitumor therapy in HCC, with HSP90 offering a novel target.
